# Determinants of Sleep Quality: A Cross-Sectional Study in University Students

**DOI:** 10.3390/ijerph20032019

**Published:** 2023-01-23

**Authors:** Johanna Marie Schmickler, Simon Blaschke, Rebecca Robbins, Filip Mess

**Affiliations:** 1 Department of Sport and Health Sciences, Technical University of Munich, 80992 Munich, Germany; 2 Division of Sleep Medicine, Harvard Medical School, Boston, MA 02115, USA

**Keywords:** Pittsburgh sleep quality index, determinants, higher education, university students

## Abstract

When entering the university setting, poor sleep quality is reportedly prevalent among students and has been linked to a range of adverse health outcomes, including reduced academic performance. Moreover, determinants of sleep quality are not yet fully understood. This study was designed to (1) assess the prevalence of poor sleep quality and (2) identify determinants of sleep quality in German university students. In total, 1,684 undergraduate and graduate students (50.6% female, mean age 22.87 ± 3.15 years) from multiple academic disciplines completed a cross-sectional online survey assessing socio-demographic, health, and study-related indicators and sleep quality using the Pittsburgh Sleep Quality Index (PSQI). In our sample, 820 (48.7%) met the PSQI cut-off score (>5) for poor sleep quality. Multiple regression analysis showed that older age, being a business student, lower subjective social status, poorer self-rated health, stress, exhaustion, and poor academic performance significantly predicted poor sleep quality. Our findings document a high prevalence of poor sleep quality among university students and suggest that business students, especially, might be exposed to a greater risk for poor sleep quality. Furthermore, the results of this study are valuable for academic staff to develop tailored interventions to promote healthy sleep-in university students.

## 1. Introduction

The university years are marked by drastic changes to living arrangements, academic responsibilities, increased social obligations, and newfound independence [1,2]. Following these transitional challenges, heightened psychological distress and reduced psychological well-being are prevalent, which are an issue of public concern [3], and they even more prevalent in university students than in their working peers [4]. It may not be surprising then that risky behaviors and decisions are common during this life stage, including poor dietary quality, weight gain, reduced physical activity [5], smoking, alcohol, and substance misuse [6].

Unfortunately, attending university is also characterized by insufficient sleep [7,8]. Between 20% and 40% of university students [7,8,9,10] are getting less sleep than what has been recommended for that age group (7 to 9 h [11]), and students report that sleep is among the first health behaviors they sacrifice during university [12]. Moreover, sleep deficiency is a routinely accepted phenomenon in higher education [13,14] and is considered a reasonable choice for students to make in order to maintain the balance of study demands [12] and social obligations [2], suggesting that social norms in universities are not conducive to sleep. The fact that most university students do not prioritize sleep leads to a deterioration in learning capacity and academic performance [15,16]. Moreover, insufficient sleep can have serious effects on students’ physical health outcomes, including obesity [17], hypertension [18], and diabetes [19]. The impact on mental health can be just as serious, and existing research recognizes the role played by poor sleep in the development of student mental health conditions, such as depression and anxiety symptoms [20,21,22,23], as well as stress and burnout [23,24].

### 1.1. Sleep Quality in University Students

For the most part, sleep duration has gained great recognition as an important sleep characteristic in earlier research, whereas more recent studies have focused on the evaluation of sleep quality. Although sleep quality and sleep duration are inextricably linked, their effects are not simply additive, and it has been proposed that sleep quality might be more closely related to health outcomes [25,26]. Despite its imprecise definition, “sleep quality” is a commonly accepted term that has been conceptualized as a construct comprised of both one’s subjective satisfaction with the sleep experience and quantitative components of sleep such as sleep duration, sleep onset latency, maintenance of sleep, and sleep efficiency [27,28]. In young adults aged 18 to 25 years, prolonged sleep privation, a sleep latency of >45 min, more than three awakenings of >5 min per night, and a sleep efficiency of less than 64% indicate poor sleep quality [28].

A considerable number of studies have been conducted among student cohorts across different socio-cultural regions [9,10,29,30,31,32,33,34,35,36,37] using the well-established Pittsburgh Sleep Quality Index [38] which identifies good and poor sleepers. Results of these studies suggest that poor sleep quality is particularly common among university students, with between 30% and 70% of students classified as poor sleepers. More recently, it has been proposed that pandemic-led restrictions were significant contributors to students′ sleep behavior, including bedtime schedules, sleep latency, and sleep duration, which caused a substantial worsening of their sleep quality [39]. Since the current literature is widely limited to US [10,30] and Chinese students [31,32,35], the findings may not reflect sleep quality rates among university students attending higher education in Europe, which has several different characteristics relating to living arrangements, tuition, enrollment, and infrastructure [40]. Additional studies are needed to characterize rates of poor sleep quality in students enrolled in European universities, particularly as the majority of previous research has examined medical [32,34] or undergraduate students [30,31,33] in other regions.

### 1.2. The Determinants of Sleep Quality in University Students

To date, the literature exploring sleep quality among university students has investigated the determinants of sleep quality, including socio-demographic characteristics, behavioral and lifestyle factors, and mental health status. Thus, previous research in student populations has indicated that sleep quality varies by sex and age, though findings are inconsistent. Some studies [9,10,36,37] found that female students reported poorer sleep quality, while others found no significant sex differences [30,31,35]. Barriers to good sleep quality in female students might be relate to a greater prevalence of reduced sleep efficiency and sleep disturbances [10,37] as well as the menstrual cycle, which affects sleep in women [41]. Increased age was found to predict poor sleep quality [35] in some but not all studies [10,30,31] and was even found to be a protective factor in a sample of Jordanian university students [29]. In some studies [10,30,31] the age range of the university students was small, making it less likely to observe age-related effects on sleep quality. Due to missing coping strategies, certain groups, including freshmen, more frequently report disturbed sleep and poor sleep quality [9]. Further, the role of student living arrangements on sleep quality is still unclear. While one study [42] suggested that students residing on their own or with their parents had a higher prevalence of sleep problems, living in a dormitory was associated with worse sleep quality in another study [43]. Evidence from cross-sectional studies indicated that students from lower socio-economic backgrounds [44] and those that perceived themselves in dire financial situations [45] were significantly more likely to report disturbed sleep and poor sleep quality. In addition, research suggests that sleep quality is lower in working students compared to non-working students [45,46]. According to the authors, these factors are in some way linked: Financial needs make it necessary for some students to take on a job while simultaneously attending university, which increases the challenge of balancing work schedules, academic workload, and sleep. Moreover, when entering the university environment, risk behaviors such as lack of physical activity [32], smoking [47], and frequent alcohol drinking [31,35] are highly prevalent and increase the likelihood of poor sleep quality in this population. Both quantitative and qualitative research has shown that students’ dissatisfaction with their academic environment and demanding workloads have been linked to poor sleep quality in this population. Hence, university students reporting dissatisfying or low academic performance were more likely to report poor sleep quality [30,32]. To meet academic workloads, first-year students participating in a qualitative interview study frequently reported staying up late, which delayed their sleep onset and collided with academic timetables [2]. Taken together, entering university can be considered a challenging phase in adolescents’ lives that is characterized by concessive strain. Thus, high levels of perceived stress have been postulated as a central factor in predicting poor sleep quality among university students [30,48].

Although numerous studies [21,29,30,31,35,47] on the determinants of sleep quality among university students have been conducted, other factors such as burnout and student engagement have been overlooked. Emerging from prolonged emotional and interpersonal stress [49], burnout has been recognized as an increasing hazard with high prevalence among university students [50]. Student burnout is commonly understood as a syndrome of emotional exhaustion induced by high perceived study demands, the development of a cynical and detached attitude towards one′s studies, and feelings of inadequacy [51]. Opposite to the construct of burnout, student engagement has been identified as the positive affective-motivational state of fulfillment [52] and is positively associated with study resources. Both constructs have been suggested to be of great importance in the university setting and are relevant determinants of students′ health and academic performance [52,53]. Previous research among students has acknowledged the association between insufficient sleep and burnout [54,55] and preliminary results on its causality suggest that burnout may increase the likelihood of poor sleep quality [24]. Further, an explorative analysis observed that high levels of student engagement are significantly positively related to the amount of sleep [55]. However, these findings are limited to cohorts of medical students, and further studies are needed to address the role of academic burnout on sleep quality among university students from other majors. So far, much uncertainty still exists on whether highly engaged students demonstrate better overall sleep quality.

### 1.3. Relevance and Aims of the current Study

Poor sleep quality is a ubiquitous phenomenon among university students, which is induced by a broad array of socio-demographic factors and health risks and is also caused by academic demands. However, current knowledge is mainly derived from studies that examined undergraduate students, focused on medical students, and is limited to distinct regions. Despite previous studies, research examining the determinants of sleep quality among university students must draw attention to study-related factors in order to provide a comprehensive overview. From a health-promoting perspective, understanding determinants is crucial to public health [56] and immanent for developing future interventions that improve sleep quality [57] in university students. Hence, the purpose of this study was to (1) investigate the prevalence of poor sleep quality among undergraduate and graduate students from various majors at a large university in Germany. We further (2) examined the determinants of sleep quality, including socio-demographic, health, and study-related indicators.

## 2. Materials and Methods

### 2.1. Procedure

The data presented in this study originated from an online survey conducted at a large university in Germany between July and August 2021. In total, N = 45,812 students were contacted via their university email addresses and invited to participate. Reminders were sent out via email circulators and posted on university-affiliated social media platforms. A link in the recruitment email connected to an anonymous online survey that was hosted using EFS Survey Unipark (Tivian XI GmbH, Cologne, Germany). Participation was voluntary, and formal consent was required from all participants. This study received full IRB approval from the local ethics committee and was conducted in accordance with the Declaration of Helsinki.

### 2.2. Sample

Of the target group, 2284 initiated participation in the survey, providing a response rate of 5%. For this study, undergraduate and graduate students aged 18 years and older were eligible, resulting in a total sample of 1684 students (age *M* = 22.87; *SD* = 3.15; range 18–50 years), of which 819 were men (48.6%), 852 were women (50.6%), and 13 indicated non-binary gender (0.8%). The majority of participants were in their fourth or above year of higher education (48.7%), and they were students from the fields of Informatics (16.8%) or Mechanical Engineering (11.4%).

Year of education was assessed as a continuous variable and manually categorized into ‘1st Year’ (including first and second semester undergraduate students), ‘2nd Year’ (including third and fourth semester undergraduate students), ‘3rd Year’ (including fifth and sixth semester undergraduate students), and ‘4th Year’ (including seven or more semester undergraduate students and students enrolled in a graduate program). Participants were asked to select their academic discipline from a list of 16 options, including ‘Aerospace and Geodesy’, ‘Architecture’, ’Business Administration’, ‘Chemistry’, ‘Civil, Geo, and Environmental Engineering’, ‘Educational Sciences’, ‘Electrical Engineering’, ‘Governance’, ‘Informatics’, ‘Life Sciences’, ‘Mathematics’, ‘Mechanical Engineering’, ‘Medicine’, ‘Physics’, ‘Sport and Health Sciences’, and ‘Other’. Discipline options reflect the study majors of the university where the data collection was conducted. Table 1 gives an overview of the socio-demographic sample characteristics.

### 2.3. Instruments

#### 2.3.1. Sleep Quality

The outcome of interest was sleep quality, assessed using the well-established Pittsburgh Sleep Quality Index (PSQI) [38]. A German PSQI version [58] was obtained from and used with the permission of the University of Pittsburgh. The PSQI is a self-administered questionnaire evaluating sleep quality and difficulties in sleep over one month (e.g., *During the past month, how often have you had trouble staying awake while driving, eating meals, or engaging in social activity?)*. The index includes a total of 19 items that are assigned to one of seven components, each weighted equally on a 0 to 3 scale: sleep quality, sleep latency, sleep duration, habitual sleep efficiency, sleep disturbances, use of sleeping medications, and daytime dysfunction. The seven component scores are summarized into a final composite global score with a range from 0 to 21. A global PSQI score above 5 indicates poor sleep quality for all age groups, with a sensitivity of 89.6% and specificity of 86.5% for identifying cases of sleep disorder [38].

#### 2.3.2. Socio-Demographic Indicators

Age was ascertained as a continuous variable. Gender was evaluated as ‘male’, ’female’, and ‘other’. Participants were asked to indicate their citizenship in an open-ended question, which was then manually grouped into ‘German’, ‘other Europe’, and ‘outside Europe’. Marital status comprised either ‘single’ or ‘married/unmarried couple’. A categorical assessment was performed for evaluating the current living situation as either ‘alone’, ‘with partner’, ‘with roommate’, or ‘with parents’. All living arrangements can be considered off-campus. Being a parent and student employment were binomially assessed (‘yes’/’no’). Monthly financial assets, after subtracting rental costs, were ascertained as a continuous variable. The MacArthur Scale (10-point scale) was used to measure perceived subjective social status (SSS) [59].

#### 2.3.3. Health Indicators

Body mass index (BMI) was handled as a continuous variable and was calculated from self-reported height and weight. Self-rated (subjective) health during the last two months was administered using a 10-point Likert scale, with top scores indicating good overall perceived health. Correspondingly, the level of physical fitness was measured using a self-reported scale from 0 to 10, with 0 indicating the lowest and 10 indicating the highest level of fitness. Smoking behavior was binominally assessed (‘yes’/‘no’). Alcohol drinking behavior was assessed using the 3-item Alcohol Use Disorders Identification Test Consumption (AUDIT-C), a reliable and valid instrument to identify alcohol misuse [60]. A total score was calculated ranging from 0 to 12, with higher numbers being associated with an increased likelihood of hazardous alcohol usage affecting one′s safety.

The Perceived Stress Scale (PSS-10) [61] was administered as a widely used self-report measure to assess the perceived stress level in adults over the past month. The scale contains 10 items, and answers are presented on a 5-point frequency scale, ranging from never (0) to very often (4). A total score was calculated, with a possible range of 0 to 40. PSS-10 outcomes were handled as a continuous variable, with higher numbers indicating a higher level of perceived stress.

#### 2.3.4. Study-Related Indicators

In this study, we used the short version of the Maslach Burnout Inventory–Student Survey (MBI-SS), which has been considered a valid measure of student burnout [62]. The 9-item questionnaire assesses student burnout as a three-dimensional construct, including (1) emotional exhaustion (e.g., *Studying or attending a class is really a strain for me*), (2) cynicism (e.g., *I have become less enthusiastic about my studies*), and (3) academic efficacy (e.g., *During my studies I do not feel confident that I am effective in getting things done*). Each item was rated on a 7-point Likert scale ranging from never (0) to always (6). For each dimension, mean scores were calculated and handled as a continuous variable, with higher scores indicating more frequent symptoms.

Student engagement was measured using the German short version of the Utrecht Work Engagement Scale, Student Version (UWES-9S), developed by Schaufeli and Bakker [63], which showed adequate psychometric properties [64]. The UWES-9S comprises three subscales with three items each reflecting the underlying dimensions of student engagement: (1) vigor (e.g., *When I am doing my work as a student, I feel bursting with energy*), (2) dedication (e.g., *My studies inspire me*), and (3) absorption (e.g., *I feel happy when I am studying intensely*). All items are rated on a seven-point frequency scale, ranging from 0 (never) to 6 (always). A total mean score across all three dimensions was calculated and used as an overall measure of student engagement, with higher values indicating higher student engagement [64].

As a measure of study performance, participants were asked whether they achieved the required semester grade points within the current semester (‘yes’/‘no’). Using a scale from 1–10, students were asked to indicate their study satisfaction, with the top referring to being completely satisfied.

#### 2.3.5. Statistical Analysis

To facilitate harmonized data quality, data cleaning procedures were employed, including the removal of ineligible cases and invalid questionnaire responses [65]. Based on the descriptive analysis of missing values, participants with more than 50% missing values were excluded (listwise deletion) under the assumption that missing values were not at random due to item nonresponse [66]. Missing data in these valid cases were handled using multiple imputation techniques by multivariate-chained equations (MICE) [67,68], generating 25 separate imputed datasets. Analyses run on each dataset were pooled according to Rubin′s rules [69]. Descriptive outcomes from continuous variables were described by means (*M*) and standard deviations (*SD*). Likewise, the outcomes from dichotomous and categorical variables were presented as frequencies and percentages.

Following previous research [9,10,30,32], we first analyzed sleep quality differences of the PSQI global mean between categorical groups using an independent samples *t*-test or analysis of variance (ANOVA) with Tukey HSD post hoc testing for more than two groups. Multiple regression analysis was performed to explore which characteristics were a significant predictor of sleep quality. Prior to the multiple regression analysis, the assumptions of linearity, normality, homoscedasticity, and independence of the residuals were tested [70]. We consulted R-squared (*R^2^*) and *p*-value to assess the goodness-of-fit (model accuracy) [71]. Standardized and unstandardized effect sizes are reported to describe the association between predictors and the outcome variable. F-statistics are reported using an imputation data set. For group differences and regression analysis, Cohen’s *d* was computed as a measure of standardized effect size, with 0.2 considered a small effect, 0.5 a medium effect, and 0.8 a large effect. [72] Two-sided *p* < 0.05 was applied to determine the statistical significance, with a confidence interval of 95%. Data analysis was performed using R and the RStudio interface (Version 4.1.2, RStudio Inc., Boston, MA, USA).

## 3. Results

### 3.1. Sleep Quality

According to the PSQI results, 820 (48.7%) out of 1,684 university students were classified as poor sleepers. On average, the PSQI global score of our sample was 6.03 ± 2.87 (Range: 0 to 19.0), which is above the cutoff for good sleepers (≤5), indicating that sleep quality was impaired.

Overall, gender had a statistically significant effect on PSQI outcomes (F(21,681) = 8.841, *p* < 0.001, $\eta_{p}^{2}$ = 0.010, *d* = 0.20). A Tukey HSD post hoc analysis showed that female students reported significantly higher mean global PSQI scores compared to male students (*p* < 0.001). Statistically significant sleep quality differences (F(31,680) = 3.804, *p* = 0.010, $\eta_{p}^{2}$ = 0.007, *d* = 0.17) were found for students’ living situations, with post hoc analysis demonstrating that students residing on their own reported significantly poorer overall sleep quality compared to students living with their parents (*p* = 0.004). Statistically significant ANOVA or samples *t*-test outcomes were not observed for a year of education (F(31,680) = 0.375, *p* = 0.771), academic discipline (F(151,668) = 1.419, *p* = 0.129), citizenship (F(21,681) = 0.152, *p* = 0.859), marital status (*t*(1682) = 0.384, *p* = 0.701), employment status (*t*(1682) = −0.264, *p* = 0.792), and being a parent (*t*(1682) = −0.297, *p* = 0.767). Table 1 outlines the descriptive results of socio-demographic indicators stratified by good and poor sleepers according to the PSQI global score.

Table 2 displays the descriptive results of health indicators stratified by good and poor sleepers according to the PSQI global score. Being a smoker was associated with statistically significant lower sleep quality compared to self-reported non-smokers (*t*(1682) = 2.79, *p* = 0.005, *d* = 0.20).

Table 3 describes the descriptive results of study-related indicators stratified by good and poor sleepers according to the PSQI global score. Better sleep quality was found in students that passed the grade point requirements compared to those that failed the requirements during the current semester (*t*(1682) = −7.85, *p* < 0.001, *d* = 0.42).

### 3.2. Predictors of Sleep Quality

A linear multiple regression analysis was performed to determine the predictors of sleep quality as the dependent variable. With our model, we independently tested the effects of socio-demographic, health, and study-related indicators that were included as either categorical or continuous variables on sleep quality. The total variance accounted for by the model was 35.9% (R^2^_adjusted_ = 0.342; F_(43, 1640)_ = 21.05; *p* < 0.001). With a small effect, age (*β =* 0.09, *t* = 2.97, *p* = 0.003, *d* = 0.28) was found to be a risk factor for poor sleep quality. Regarding the academic disciplines, being a business student (*β =* 0.35, *t* = 2.24, *p* = 0.026, *d* = 0.87) was the central predictor for sleep quality in our model, indicating an increased risk for poor sleep quality. Further, subjective social status (*β =* −0.06, *t* = −2.44, *p* = 0.015, *d* = 0.22) with a small effect and self-rated health (*β =* −0.22, *t* = −7.63, *p* < 0.001, *d* = 0.56) with a medium effect negatively predicted sleep quality and can be considered protective against poor sleep quality. Medium effects were also observed for perceived stress (*β =* 0.27, *t* = 8.31, *p* < 0.001, *d* = 0.68), which can be considered a risk factor for poor sleep quality. Finally, exhaustion (*β =* 0.16, *t* = 5.02, *p* < 0.001, *d* = 0.43) and academic performance (*β =* 0.10, *t* = 2.08, *p* = 0.038, *d* = 0.30), independently and significantly, predicted sleep quality with small effects and can be considered risk factors for poor sleep quality. Table 4 summarizes the significant results of the regression analysis. Complete regression analysis results are presented in Appendix A (Table A1).

## 4. Discussion

### 4.1. Prevalence of Poor Sleep Quality

The first objective of this study was to examine the prevalence of poor sleep quality among undergraduate and graduate students at a large university in Germany. With the cutoff (PSQI > 5), the descriptive analysis showed a PSQI global score of 6.03 ± 2.87 and a 49% prevalence of poor sleep quality. Thus, our findings are in between the prevalence results from earlier research [10,29,31,32,33] using the same methodology and showing that 30–70% of the students were classified as poor sleepers. The majority of these studies on sleep quality in university students has been conducted across different countries, including Jordan [29], the U.S. [10], Mongolia [32], China [31], and Ethiopia [33]. While these studies emphasize the high prevalence of poor sleep quality among university students, comparisons with the present study remain difficult due to the socio-demographic and higher education characteristics of different cultural regions. Using a different methodology, earlier research conducted by Schlarb et al. [9] examined sleep disturbances in a sample of university students from Luxembourg and Germany. According to their classification, 48% of students were identified as bad sleepers with a PSQI cut-off score of >5, and 18% had severe sleep problems according to the PSQI (cut-off > 10). When combining both categories in the German student population, poor sleep quality (59%) was more prevalent than in our sample (49%). Overall, a higher mean age and a greater proportion of female participants were reported in their sample, which might explain the discrepancies between prevalence outcomes [9,10,35].

Moreover, we want to highlight the fact that comparisons to earlier studies may be somewhat limited. To the best of our knowledge, this is the first study to present data on sleep quality that was collected in the early stages of normalization after the COVID-19 pandemic-led restrictions among undergraduate and graduate students in Germany. In their systematic review, Valenzuela et al. [39] examined the impact of the COVID-19 outbreak on sleep among undergraduate students from different countries. While several studies demonstrated that pandemic-related lockdowns caused a substantial worsening of sleep quality [73,74,75], especially among female students, improved sleep quality was reported in another study on performing arts students [76]. Considering that pandemic-led restrictions were significant contributors to students′ sleep behavior, including bedtime schedules, sleep latency, and sleep duration [39], further studies are needed to investigate whether students′ sleep will return to normalcy or a new normal.

### 4.2. Determinants of Sleep Quality

Given the high prevalence of poor sleep quality and its aforementioned serious health implications [15,16,17,18,19] among student populations, increased awareness of the importance of sufficient sleep is needed. To meet these demands, the present study was designed to identify socio-demographic, health, and study-related indicators, providing a more complete understanding of the determinants of sleep quality in this population.

#### 4.2.1. Socio-Demographic Indicators

Regarding socio-demographic variables, prior literature on university students has observed inconsistencies regarding the role of gender and age on sleep quality outcomes [9,10,29,30,31,35]. According to our regression analysis, gender is not a predictor for sleep quality, which is in line with results from earlier studies [30,31,35]. Although with a small effect, we found older age to be a significant risk factor for poor sleep quality, which was also observed in another study [35]. The results of the present study are contrary to studies [10,30,31] that did not find age to be associated with sleep quality, which may be attributable to the narrow age range of their student populations. Notably, the majority of students in the current study were in their fourth (or above) year of education and therefore presented a higher mean age as documented in other studies [10,30,31]. While it can be suggested that older students face additional pressures, including increased study demands due to imminent graduation and career responsibilities, they might more frequently neglect their sleep compared to younger students. In addition, poorer sleep quality in older students might also be related to the fact that these students more often work part-time while studying and are already parents compared to younger students. Thus, the present study raises the possibility that being an older student may pose risks for poor sleep quality. Additional studies with longitudinal designs are needed to examine sleep patterns in both undergraduate and graduate students by simultaneously assessing underlying factors such as health behaviors and academic demands to present further insights into the role of age on sleep quality. In line with earlier findings [44], our study among German university students identified lower social status as a risk factor for poor sleep quality.

Besides other determinants, the current study examined the role of different academic disciplines on sleep quality, which has been largely neglected in previous studies. In fact, with a large effect, our model showed that being a business student is associated with a significantly higher risk for poor sleep quality compared to architecture students that served as the reference group due to dummy coding. While earlier research has emphasized the high prevalence of poor sleep quality among medical students [32,34], our regression results suggest that business students may be more likely to be poor sleepers. Similar conclusions have been drawn from an earlier study examining mental distress, alcohol consumption, and help-seeking behavior among medical and business students [77]. Compared to medical students, higher levels of stress and disengagement, as well as greater alcohol consumption, a higher perceived academic workload, and increased exhaustion, were observed among business students. The authors suggested that, even though stress levels in medical students are high, they may not be more stressed than students from other academic disciplines. Further, preliminary research on health behaviors and mental health characteristics across different academic disciplines might help to elucidate the findings from the present study. For instance, one study examined health-related behavioral clusters in Irish university students, and among others, business students were significantly more likely to be assigned to a cluster characterized by low physical activity and poor nutritional behavior [78]. Moreover, business students appear to have characteristic personality traits that might impact their sleep quality: When compared to non-business students, business students were more sociable, outgoing, and talkative, as well as tough-minded and hard-working [79]. Thus, one may hypothesize that business students are not only more likely to engage in social activities involving staying up late and consuming alcohol but also more likely to sacrifice their sleep to meet academic demands, which ultimately might provoke poor sleep quality.

Despite the lack of research on sleep behaviors across academic disciplines, the literature highlights the fact that business students are at risk of developing adverse health-related behaviors. Although speculative at this point, it is possible that sufficient sleep and good sleep quality are not being prioritized among these students, which could explain the findings of our study indicating that business students are at risk for poor sleep quality.

#### 4.2.2. Health-Related Indicators

Further analysis regarding health-related indicators revealed that better self-rated health predicts sleep quality. Our results reflect earlier research in the general population examining the positive association between sleep quality and self-rated health [80]. Corresponding to what has been reported in a study among older adults [81], the current study contributes to the body of knowledge indicating that self-rated health is an important predictor of sleep quality among university students. In our regression model, we found that perceived stress was a significant predictor of sleep quality. These results are consistent with previous studies [30,48], indicating that high levels of perceived stress put university students at risk for poor sleep quality. Entering the university setting is characterized by demanding factors such as academic workload, social responsibilities, and irregular schedules, which are accompanied by insufficient coping strategies that provoke stress-induced sleep difficulties [82]. Our results add to the body of previous studies that call for interventions that include elements of stress management and build upon existing coping strategies to address poor sleep patterns in university students [83,84].

#### 4.2.3. Study-Related Indicators

The current study examined the role of study-related indicators, including student burnout, study satisfaction, and academic performance, on sleep quality. Among the three burnout dimensions [51] that were separately analyzed in our regression model, only emotional exhaustion was identified as a statistically significant predictor of poor sleep quality. This is contradictory to the findings from Pagnin et al. [24], which evaluated the relationship between burnout and sleep disorders in medical students. The authors found that neither emotional exhaustion, cynicism, nor academic efficacy predicted sleep quality, but emotional exhaustion was shown to increase the chances for daytime sleepiness. However, recent findings from a longitudinal study of workers indicated that work-related exhaustion, rather than other burnout dimensions, was a risk factor for sleep difficulties [85]. In this study, the authors suggested that exhaustion can be related to dysfunctional stress responses and pre-sleep arousal, which are followed by increased difficulties initiating sleep and shorter sleep duration [86]. Thus, there is abundant room for further studies among student cohorts to shed light on the relationship between burnout dimensions and sleep quality. Lastly, we were able to show that students reporting failing the grade-point requirements were more likely to be classified as poor sleepers compared to students that meet class requirements. Although previous studies used different measures to ascertain academic performance, our results are supported by those observations [30,32]. Considering that poorer academic performance may require students to pull an all-nighter to meet academic demands, this might result in a vicious cycle of sleep deprivation and poor sleep quality.

### 4.3. Strengths and Limitations

This study has several implications and strengths. Our study adds to the existing knowledge [9,10,30,31,32,33,35] by providing a deeper understanding of the determinants of sleep quality in university students from various academic disciplines using validated questionnaires. Thus, these findings can be applied to a broader student context and contribute to the current literature by examining German students. In contrast to the majority of previous studies [9,10,30], male students are equally represented in our cohort, and we also included graduate students from various study disciplines. Finally, the fact that some study disciplines may experience greater chances for poor sleep quality has important practical implications for higher education and stresses the implicit need for university counselors to conceptualize targeted interventions and sleep promotion.

Despite the significance of the results, this study has limitations. First, participants in the current study were exclusively recruited from a large university in Germany, which is not representative of all students in Germany. Hence, this limits the generalizability of our findings. Furthermore, the cross-sectional design of the present study prevents elucidating causality between study variables. Thus, we propose the use of longitudinal studies tracking students′ health behaviors, study demands and resources, as well as sleep patterns across several years of studying and academic disciplines to uncover causalities and possible dynamic relationships between variables. Further studies might also benefit from incorporating objective methods such as actigraphy or qualitative methods using semi-structured interviews and sleep diaries to allow for capturing insights that may have been missed due to the self-reported measures used in the present study [87]. Lastly, the response rate of 5% was lower than corresponding estimates in previous studies [10,29,30,31,33] but similar to observations in an earlier study in European students [9]. However, the low response rate must be recognized as a limitation of our study. Several reasons might be associated, including the comprehensive nature of the online survey, with >116 items requiring 20 min for completion. Higher response rates might be attributable to incentives for participation such as class credits and monetary prices, as have been provided in other research [10,30], but not in the present study. In addition to recruitment via email and social media, an extensive oral notification of the study from teaching staff might serve as a useful tool to increase the number of participants in future studies.

## 5. Conclusions

Our study extends the current literature by providing empirical support for the current concerns regarding the prevalence of poor sleep quality among university students from Germany. Further, this study gives a broad insight into the determinants of sleep quality. With a large effect, this study was able to uncover that business students might face a greater risk of poor sleep quality. Moreover, with medium effects, we showed that poorer self-rated health and higher levels of perceived stress increase the likelihood of poor sleep quality. In tackling the high prevalence of poor sleep quality, several measures are recommended. University health education programs should deliberately address the importance of sleep as well as the relationship between health behaviors and sleep quality, particularly targeting business students. In addition, more effort should be made to address the facets that underlie students’ poor sleep quality. For example, it may be crucial to support students that are struggling with appropriate strategies that allow them to arrange their limited time and resources for a better balance between sufficient sleep, health behaviors, social interactions, and academic demands. Academic staff may also consider adjusting curricular workloads and accommodating students with flexible schedules to reduce study-related distress. Finally, our findings are of great value for the conceptualization of future sleep-promoting programs that aim at maintaining a healthy study-work-life balance, good sleep habits, appropriate stress, and time management.

## Figures and Tables

**Table 1:** Descriptive characteristics of socio-demographic indicators stratified by PSQI classification.

Variables	Total Sample (*N* = 1684)	Total PSQI	PSQI ≤ 5 (*n* = 864)	PSQI > 5 (*n* = 820)
**Age**, years	22.87 ± 3.15 (18.0–50.0)		22.74 ± 3.14	23.01 ± 3.15
**Gender**				
Male	819 (48.6%)	5.74 ± 2.73	448 (54.7%)	371 (45.3%)
Female	852 (50.6%)	6.32 ± 2.97	407 (47.8%)	445 (52.2%)
Other	13 (0.8%)	5.62 ± 3.18	9 (69.2%)	4 (30.8%)
**Year of Education**				
1st	347 (20.6%)	6.10 ± 2.85	179 (51.6%)	168 (48.4%)
2nd	272 (16.2%)	6.10 ± 2.75	129 (47.4%)	143 (52.6%)
3rd	244 (14.5%)	5.87 ± 2.77	130 (53.3%)	114 (46.7%)
4th +	821 (48.8%)	6.02 ± 2.95	426 (51.9%)	395 (48.1%)
**Academic Discipline**				
Aerospace and Geodesy	30 (1.8%)	5.67 ± 2.64	19 (63.3%)	11 (36.7%)
Architecture	45 (2.7%)	6.09 ± 2.70	21 (46.7%)	24 (53.3%)
Business Administration	126 (7.5%)	6.38 ± 3.07	56 (44.4%)	70 (55.6%)
Chemistry	69 (4.1%)	6.19 ± 2.52	32 (46.4%)	37 (53.6%)
Civil, Geo and Environmental Engineering	110 (6.5%)	5.82 ± 2.69	60 (54.5%)	50 (45.5%)
Educational Sciences	40 (2.4%)	5.55 ± 2.85	23 (57.5%)	17 (42.5%)
Electrical Engineering	123 (7.3%)	5.73 ± 2.74	67 (54.5%)	56 (45.5%)
Governance	20 (1.2%)	4.80 ± 1.70	11 (55.0%)	9 (45.0%)
Informatics	283 (16.8%)	6.01 ± 2.94	149 (52.7%)	134 (47.3%)
Life Sciences	140 (8.3%)	6.61 ± 2.79	63 (45.0%)	77 (55.0%)
Mathematics	61 (3.6%)	6.69 ± 4.04	30 (49.2%)	31 (50.8%)
Mechanical Engineering	192 (11.4%)	5.78 ± 2.77	100 (52.1%)	92 (47.9%)
Medicine	137 (8.1%)	6.02 ± 2.98	74 (54.0%)	63 (46.0%)
Physics	87 (5.2%)	6.31 ± 2.69	36 (41.4%)	51 (58.6%)
Sport and Health Sciences	137 (8.1%)	5.85 ± 2.66	77 (56.2%)	60 (43.8%)
Other	84 (5.0%)	5.99 ± 2.81	46 (54.8%)	38 (45.2%)
**Citizenship**				
German	1451 (86.2%)	6.02 ± 2.84	751 (51.8%)	700 (48.2%)
Other Europe	160 (9.5%)	5.99 ± 3.12	81 (50.6%)	79 (49.4%)
Outside Europe	73 (4.3%)	6.21 ± 2.98	32 (43.8%)	41 (56.2%)
**Marital Status**				
Single	1316 (78.1%)	6.04 ± 2.88	666 (50.6%)	651 (49.4%)
Married/unmarried couple	368 (21.9%)	5.98 ± 2.84	183 (49.7%)	184 (50.3%)
**Living Situation**				
Alone	382 (22.7%)	6.41 ± 2.86	179 (46.9%)	203 (53.1%)
With partner	231 (13.7%)	6.08 ± 2.86	113 (48.9%)	118 (51.1%)
With roommate	540 (32.1%)	6.00 ± 2.97	280 (51.9%)	260 (48.1%)
With parents	531 (31.5%)	5.77 ± 2.75	292 (55.0%)	239 (45.0%)
**Being a Parent**				
Yes	17 (1.0%)	5.82 ± 3.07	7 (41.2%)	10 (58.8%)
No	1667 (99.0%)	6.03 ± 2.87	857 (51.4%)	810 (49.6%)
**Student Employment**				
Yes	911 (54.1%)	6.01 ± 2.87	462 (50.7%)	449 (49.3%)
No	773 (45.9%)	6.05 ± 2.86	402 (52.0%)	371 (48.0%)
**Monthly available Assets, €**	4834.24 ± 392.85 (0–7000)		498.81 ± 420.66	468.88 ± 360.86
**Subjective Social Status**	6.29 ± 1.80 (1.0–10.0)		6.55 ± 1.74	6.01 ± 1.82

Notes. *N* = 1684; *PSQI* Pittsburgh Sleep Quality Index; *M* Mean; *±SD* Standard deviation; Range (*Minimum*–*Maximum*).

**Table 2:** Descriptive characteristics of health indicators stratified by PSQI classification.

Variables	Total Sample (*N* = 1684)	Total PSQI	PSQI ≤ 5 (*n* = 864)	PSQI > 5 (*n* = 820)
**BMI, kg/m^2^**	22.18 ± 3.29 (14.0–60.2)		22.11 ± 3.24	22.26 ± 3.34
**Self-Rated Health**	5.92 ± 2.40 (1.0–10.0)		6.95 ± 2.07	4.84 ± 2.24
**Perceived Level of Fitness**	6.72 ± 1.86 (1.0–10.0)		7.11 ± 1.71	6.30 ± 1.93
**Smoking**				
Yes	224 (13.3%)	6.53 ± 2.91	94 (42.0%)	130 (58.0%)
No	1460 (86.7%)	5.95 ± 2.86	770 (52.7%)	690 (47.3%)
**Alcohol Use**	3.83 ± 2.00		3.84 ± 2.02	3.82 ± 1.97
**Perceived Stress**	20.97 ± 7.34 (1.0–40.0)		17.57 ± 6.72	24.38 ± 6.23

Notes. *N* = 1684; *PSQI* Pittsburgh Sleep Quality Index; *BMI* Body-Mass-Index; *M* Mean; *±SD* Standard deviation; Range (*Minimum*–*Maximum*).

**Table 3:** Descriptive characteristics of study-related indicators stratified by PSQI classification.

Variables	Total Sample (*N* = 1684)	Total PSQI	PSQI ≤ 5 (*n* = 864)	PSQI > 5 (*n* = 820)
**Student Burnout**				
Exhaustion	3.03 ± 1.66 (0.0–6.0)		2.34 ± 1.50	3.76 ± 1.51
Cynicism	1.70 ± 1.72 (0.0–6.0)		1.26 ± 1.50	2.17 ± 1.82
Efficacy	2.35 ± 1.69 (0.0–6.0)		1.81 ± 1.49	2.92 ± 1.70
**Student Engagement**	2.99 ± 1.09 (0.0–6.0)		3.23 ± 1.07	2.75 ± 1.06
**Academic Performance**				
Passed grade point requirements	1193 (70.8%)	5.68 ± 2.65	672 (56.3%)	521 (43.7%)
Failed grade point requirements	491 (29.2%)	6.87 ± 3.19	192 (39.1%)	299 (60.9%)
**Study Satisfaction**	7.67 ± 2.04 (1.0–10.0)		8.02 ± 1.88	7.31 ± 2.13

Notes. *N* = 1684; *PSQI* Pittsburgh Sleep Quality Index; *M* Mean; *±SD* Standard deviation; Range (*Minimum*–*Maximum*).

**Table 4:** Significant results of multiple regression analysis with sleep quality as the dependent variable.

Predictors	Unstandardized Coefficients		Standardized Coefficients	*t*	*p*	95% CI
	B	Std. Error	*β*			
**Age**	0.08	0.03	0.09	2.97	0.003 **	[0.03; 0.15]
**Academic Discipline**						
Business Administration	0.99	0.45	0.35	2.24	0.026 *	[0.04; 0.66]
**Subjective Social Status**	−0.09	0.04	−0.06	−2.44	0.015 *	[−0.08; 0.01]
**Self-rated Health**	−0.27	0.04	−0.22	−7.63	<0.001 ***	[−0.28; −0.17]
**Perceived Stress**	0.11	0.01	0.27	8.31	<0.001 ***	[0.21; 0.34]
**Student Burnout**						
Exhaustion	0.28	0.06	0.16	5.02	<0.001 ***	[0.10; 0.23]
**Academic Performance**						
Failed grade point requirement	0.30	0.14	0.10	2.08	0.038 *	[0.006; 0.20]

Notes. * *p* < 0.05, ** *p* < 0.01 (two-tailed), *** *p* < 0.001 (two-tailed); 95% CI = 95% confidence interval; academic discipline = reference group is Architecture due to dummy coding. Insignificant findings are not displayed in this table but can be found in Appendix A.

## Data Availability

The datasets used and analyzed in the current study are available in a highly anonymized form from the corresponding author upon a reasonable request.

## Appendix A

**Table A1:** Multiple regression results with sleep quality as the dependent variable.

Predictors	Unstandardized Coefficients		Standardized Coefficients	*t*	*p*	95% CI
	B	Std. Error	*β*			
**(Intercept)**	2.77	1.32	−0.21	2.10	0.036 *	[−0.75; 0.34]
**Age, years**	0.08	0.03	0.09	2.97	0.003 **	[0.03; 0.15]
**Gender**						
Male	—	—	—	—	—	—
Female	0.15	0.15	0.05	1.06	0.290	[−0.05; 0.15]
Other	−0.18	0.70	−0.06	−0.26	0.796	[−0.54; 0.42]
**Year of Education**						
1st	—	—	—	—	—	—
2nd	−0.07	0.20	−0.03	−0.37	0.710	[−0.16; 0.11]
3rd	−0.09	0.21	−0.03	−0.42	0.677	[−0.18; 0.11]
4th+	−0.17	0.20	−0.06	−0.85	0.393	[−0.19; 0.08]
**Academic Discipline**						
Aerospace and Geodesy	0.34	0.59	0.12	0.57	0.567	[−0.29; 0.52]
Architecture	—	—	—	—	—	—
Business Administration	0.99	0.45	0.35	2.24	0.026 *	[0.04; 0.66]
Chemistry	0.84	0.48	0.29	1.73	0.085	[−0.04; 0.62]
Civil, Geo and Environmental Engineering	0.56	0.45	0.19	1.22	0.222	[−0.12; 0.51]
Educational Sciences	0.22	0.54	0.08	0.40	0.688	[−0.30; 0.45]
Electrical Engineering	0.32	0.44	0.11	0.72	0.473	[−0.19; 0.42]
Governance	−0.11	0.66	−0.04	−0.17	0.868	[−0.49; 0.42]
Informatics	0.57	0.42	0.20	1.37	0.171	[−0.09; 0.48]
Life Sciences	0.66	0.43	0.23	1.52	0.129	[−0.07; 0.53]
Mathematics	0.76	0.50	0.27	1.54	0.125	[−0.07; 0.61]
Mechanical Engineering	0.30	0.42	0.11	0.71	0.476	[−0.19; 0.40]
Medicine	0.77	0.43	0.27	1.81	0.071	[−0.02; 0.56]
Physics	0.61	0.46	0.21	1.31	0.190	[−0.11; 0.53]
Sport and Health Sciences	0.64	0.44	0.22	1.47	0.143	[−0.08; 0.52]
Other	0.58	0.47	0.20	1.25	0.213	[−0.12; 0.53]
**Citizenship**						
German	—	—	—	—	—	—
Other Europe	0.21	0.23	0.07	0.92	0.359	[−0.09; 0.23]
Outside Europe	−0.24	0.34	−0.08	−0.70	0.483	[−0.31; 0.15]
**Marital Status**						
Single	—	—	—	—	—	—
Married/unmarried couple	−0.21	0.16	−0.07	−1.30	0.194	[−0.19; 0.04]
**Living Situation**						
Alone	—	—	—	—	—	—
With partner	−0.18	0.23	−0.06	−0.77	0.442	[−0.22; 0.10]
With roommate	−0.15	0.16	−0.05	−0.92	0.358	[−0.16; 0.06]
With parents	−0.25	0.17	−0.09	−1.47	0.143	[−0.20; 0.03]
**Being a Parent**						
Yes	—	—	—	—	—	—
No	0.49	0.66	0.17	0.75	0.455	[−0.28; 0.62]
**Student Employment**						
Yes	—	—	—	—	—	—
No	−0.08	0.13	−0.03	−0.65	0.515	[−0.12; 0.06]
**Monthly available Assets**, €	−0.0002	0.0001	−0.03	−1.45	0.147	[−0.12; 0.06]
**Subjective Social Status**	−0.09	0.04	−0.06	−2.44	0.015 *	[−0.08; 0.01]
**BMI, kg/m^2^**	−0.03	0.02	−0.03	−1.50	0.135	[−0.08; 0.01]
**Self-rated Health**	−0.27	0.04	−0.22	−7.63	<0.001 ***	[−0.28; −0.17]
**Perceived Level of Fitness**	0.003	0.04	0.002	0.09	0.927	[−0.05; 0.05]
**Smoking**						
Yes	—	—	—	—	—	—
No	−0.33	0.18	−0.12	−1.81	0.071	[−0.24; 0.01]
**Alcohol use**	0.01	0.04	0.008	0.33	0.744	[−0.04; 0.01]
**Perceived Stress**	0.11	0.01	0.27	8.31	<0.001 ***	[0.21; 0.34]
**Student Burnout**						
Exhaustion	0.28	0.06	0.16	5.02	<0.001 ***	[0.10; 0.23]
Cynicism	0.07	0.06	0.04	1.15	0.252	[−0.03; 0.12]
Efficacy	−0.03	0.06	−0.02	−0.62	0.536	[−0.09; 0.04]
**Student Engagement**	0.14	0.09	0.05	1.61	0.109	[−0.01; 0.12]
**Academic Performance**						
Passed grade point requirements	—	—	—	—	—	—
Failed grade point requirements	0.30	0.14	0.10	2.08	0.038 *	[0.006; 0.20]
**Study Satisfaction**	0.02	0.04	0.02	0.57	0.566	[−0.04; 0.08]

Notes. * *p* < 0.05, ** *p* < 0.01 (two-tailed), *** *p* < 0.001 (two-tailed); 95% CI = 95% confidence interval; *BMI* Body-Mass-Index; academic discipline = reference group is Architecture due to dummy coding.
